# Acute Non-infectious Cystitis Secondary to Immune-Related Adverse Events in a Patient Receiving Pembrolizumab for Treatment of Non-small Cell Lung Cancer: A Case Report

**DOI:** 10.7759/cureus.55666

**Published:** 2024-03-06

**Authors:** Leena Alhusari, Mahmoud Abdallah, Kemnasom Nwanwene, Mina Shenouda

**Affiliations:** 1 Internal Medicine, Marshall University Joan C. Edwards School of Medicine, Huntington, USA; 2 Hematology and Medical Oncology, Marshall University Joan C. Edwards School of Medicine, Huntington, USA

**Keywords:** refractory hematuria, non-bacterial cystitis, immune-related-adverse-events, pembrolizumab side effect, oncology

## Abstract

Immune-related adverse events (IrAEs) involving the bladder are seldom reported and tend to be overlooked by oncologists. Cystitis caused by immune checkpoint inhibitors (ICIs) is rarely reported, with only four documented instances in the literature, of which just one case is attributed to pembrolizumab. We present a rare occurrence of pembrolizumab-induced hemorrhagic cystitis in a 71-year-old male with stage II-b lung adenocarcinoma with an chronic indwelling Foley catheter. He presented with persistent hematuria despite the completion of a course of antibiotics for a urinary infection; a cystoscopic examination was also normal. Drug-induced cystitis was suspected and the patient was treated with prednisone as well as temporary discontinuation of pembrolizumab, which was followed by an improvement of symptoms.

## Introduction

The utilization of immune checkpoint inhibitors (ICIs) enhanced the five-year survival rate of non-small cell lung cancer (NSCLC) by approximately 5-10% [1,2]. ICIs function by stimulating the immune system's anti-tumor responses through the inhibition of immune checkpoints like cytotoxic T lymphocyte antigen-4 (CTLA-4) and programmed death-1 (PD-1), or its ligand PD-L1. These drugs augment immune activity, leading to heightened immunity against healthy organs and causing distinct toxicity compared to standard chemotherapy or other biological agents [3]. Among the observed side effects, skin, and gastrointestinal adverse reactions were the most frequently reported [4]. In this case report, we present a situation where cystitis developed following the administration of pembrolizumab, which was classified as an immune-related adverse event (irAE). Written informed consent was diligently obtained from the participant, signifying his voluntary agreement and understanding of the terms outlined in the consent document for this case report.

## Case presentation

Herein, we present a 71-year-old gentleman with a history of stage II-b lung adenocarcinoma (negative for expression of PD-L1, ALK, EGRD, and Ross) diagnosed six months back. He received four cycles of carboplatin/ pemetrexed/ pembrolizumab and then switched to maintenance pembrolizumab; his last dose of the fourth cycle of pembrolizumab was one week prior to admission. He also has benign prostatic hyperplasia (BPH) causing a urinary obstruction which was planned to be treated with prostate reduction surgery; in the meantime, the patient was equipped with an indwelling Foley catheter. He presented to the internal medicine clinic for worsening gross hematuria that began one week ago. His urine had a dark red appearance and urine analysis showed red blood cells (RBCs) 51-100/HPF, white blood cells (WBCs) 0-4/HPF, a small amount of leukocyte esterase, and negative nitrite. He completed a course of antibiotics as a treatment for a urinary tract infection. His hematuria did not resolve. He also reported feeling weak and out of breath. He denied having symptoms such as nausea, vomiting, fever, or bleeding from other parts of the body. His Foley catheter is scheduled for a change every month and was changed three weeks prior to admission. He denied a history of kidney stones and was not taking any blood thinners. On examination, his vital signs showed tachycardia with a heart rate of 117 beats per minute. Bloody urine was noticed in the urine bag; the rest of the vitals and examination were unremarkable.

The patient was admitted to the hospital for further management of his hematuria. His hemoglobin levels dropped from 8 to 6.7 (normal range 13-19 gm/dL), requiring a blood transfusion. Other laboratory findings were significant for leukocytosis, normal INR, and kidney function tests (Table 1).

**Table 1:** Laboratory values upon admission

Test	Lab result	Reference range
White cell count	15	4.5-10 × 10^9/L
Hemoglobin	6.7	11-18 × 10^9/L
Platelets	548	150-440 × 10^9/L
Blood urea nitrogen (BUN)	17	5-18 × 10^9/L
Creatinine	0.7	0.7-1.4 × 10^9/L
INR	0.98	1.3-1.7 gm/dL

**Figure 1:**
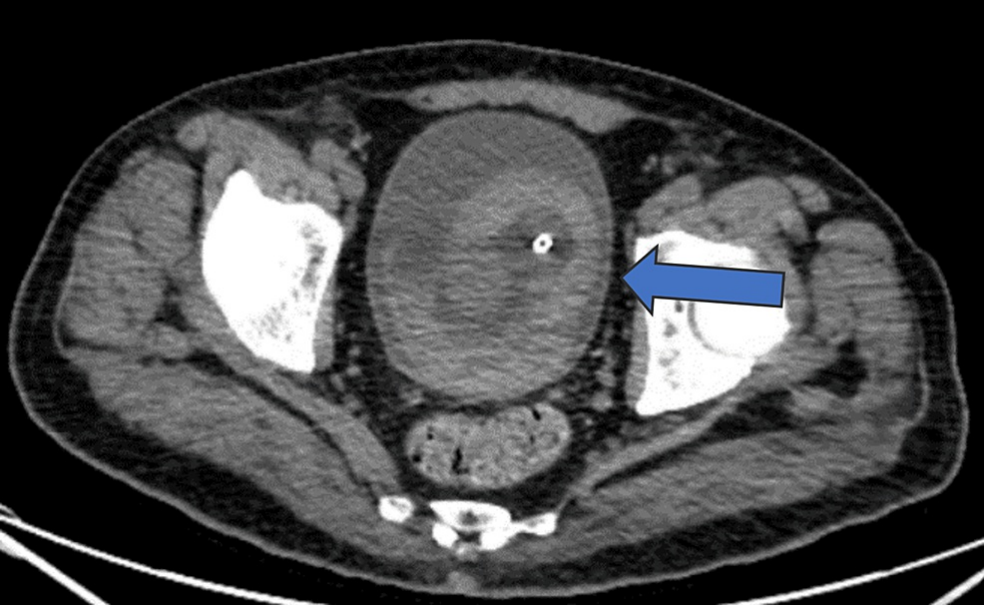
a CT scan of the abdomen and pelvis showing a sizable bladder hematoma

A CT scan of the abdomen and pelvis revealed a sizable hematoma in the urinary bladder with the Foley catheter in place, but no reported pathology in the kidneys or ureters (Figure 1). Antibiotic treatment was started based on urology recommendations given the possibility of bacterial cystitis (Table 2). However, the patient’s symptoms did not get better. The patient then underwent a cystoscopy that showed normal urethra, prostate, and bladder structure, no masses or bleeding artery; during this procedure, a supra-pubic catheter was also inserted. After a multidisciplinary consultation including the urology and oncology departments, immune-related cystitis was considered; treatment with prednisone started at 1 mg/kg. Patients also received trimethoprim-sulfamethoxazole (TMPSMX) for *Pneumocystis jirovecii* (PCP) prophylaxis.

**Table 2:** Urine culture result and susceptibility MIC: minimum inhibitory concentration

Citrobacter freundii complex	Drug MIC Interpretation	MIC Dilutional
Amikacin	S	<=16
Amoxicillin/clavulanate	R	16/8
Ampicillin	R	>16/8
Ampicillin/sulbactam	R	>16/8
Aztreonam	S	<=4
Cefazolin	R	>16
Cefepime	S	<=2
Cefotaxime	S	<=2
Ceftriaxone	S	<=1
Levofloxacin	S	<=2
Meropenem	S	<=1
Nitrofurantoin	S	<=32
Piperacillin/tazobactam	S	S<=8

During a hospital discharge follow-up appointment, the patient reported improvement in the hematuria. The primary oncologist started tapering off the steroid. Shortly, the PET scan showed evidence of disease progression and he started systemic therapy with pemetrexed and carboplatin.

## Discussion

Our report demonstrates a case of acute non-bacterial cystitis resulting from an IrAE caused by the ICI pembrolizumab. Upon reviewing the available literature, we found five reported cases of ICI-induced cystitis, with only one of those cases specifically associated with pembrolizumab [5].

The sole reported case of immune-related cystitis induced by pembrolizumab was described by Ueki et al [5]. It involved a 78-year-old female who had completed 17 cycles of pembrolizumab for the treatment of non-small cell lung cancer (NSCLC). The patient experienced symptoms consistent with increased urinary frequency and dysuria. The diagnosis of immune-related cystitis was based on histopathological findings, which revealed the presence of CD8-positive and/or TIA-1 cytotoxic granule-associated RNA binding protein-positive lymphocytes and PD-L1 expression in the urothelium. The patient's subjective symptoms and cystoscopy findings improved significantly following the initiation of prednisolone. Comparing this to our case, the diagnosis of drug-induced cystitis was made based on the significant clinical improvement with the initiation of steroid therapy. In our case, cystoscopic examination was normal and tissue biopsy was not performed.

In addition, there have been three reported cases of non-bacterial cystitis induced by nivolumab. Two of these cases were described by Shimatani et al [6]. The first patient was a 50-year-old male who received seven cycles of nivolumab for NSCLC, while the second patient was a 60-year-old male who received 12 cycles of nivolumab. Both patients developed dysuria and exhibited nonbacterial pyuria that did not respond to antibiotic treatment, with urine cultures remaining negative. The symptoms of the first case improved after discontinuation of the ICI and a course of prednisone (1 mg/kg/day), whereas the symptoms of the second case improved with discontinuation of the ICI alone. Another case described by Ozaki et al. [7] involved a patient who completed four cycles of nivolumab and presented with hematuria and dysuria. The diagnosis of IrAE cystitis was established through exclusion following a bladder biopsy. The patient's symptoms improved after receiving steroid pulse therapy with methylprednisolone (500 mg for three days), followed by maintenance prednisolone (0.5 mg/kg/day) (Table 2). By reviewing the literature, there was a single reported case of non-infectious cystitis related to the use of PD-1 inhibitor, sintilimab [8].

**Table 3:** Five reported cases of ICI-induced non-bacterial cystitis ICI: immune checkpoint inhibitor; SCC: squamous cell carcinoma

Author, year	Patient	Symptoms	Objective findings	Primary disease	ICIs (cycles)	Treatment	Pathological features
Shimatani et al., 2018 [6])	50 y/o, male	Dysuria, increased urinary frequency	Pyuria-negative urine culture	Lung SCC	Nivolumab (7)	Prednisolone (1 mg/kg/day) and discontinuation of ICI	None
	60 y/o, male		Pyuria-negative urine culture	Lung SCC	Nivolumab (12)	Discontinuation of ICI	None
Ozaki et al., 2017 [7]	62 y/o, male		Microhematuria and pyuria cystoscopy: diffuse redness; erosion urine cytology: negative	Lung SCC	Nivolumab (3)	Methylprednisolone 500 mg x 3 days followed by prednisolone (0.5 mg/kg/day) and discontinuation of ICI	Epithelial desquamation and edematous changes
Ueki et al., 2020 [5]	78 y/o, female		Microhematuria and pyuria cystoscopy: diffuse redness; erosion urine cytology: negative	Lung adenocarcinoma	Pembrolizumab (17)	Prednisolone (0.5 mg/kg/day) and discontinuation of ICI	PD-L1+ urothelial cells and CD8+ and/or TIA-1+ infiltrating lymphocytes
Present case	71 y/o, male	Gross hematuria	Pyuria, gross hematuria, negative urine culture cystoscopy: unremarkable	Lung adenocarcinoma	Pembrolizumab (4)	Prednisone (1 mg/kg/day) and discontinuation of ICI	None

ICI works effectively through the disinhibition of T cell cytotoxicity to tumors. Those augmented T lymphocytes react against healthy tissues targeting specific antigens that are still unknown. Further identification of those antigens can introduce treatment options for IrAEs that improve patient outcomes and quality of life during treatment.

The diagnosis of IrAE is by exclusion after monitoring the clinical response to managing the other common etiologies of such symptoms. Also, obtaining tissue biopsies has been reported to aid in establishing the diagnosis of IrAEs. However, due to the rarity of these events and the continuous evolution of IrAEs associated with newly introduced ICIs, well-established diagnostic and therapeutic guidelines are still lacking.

The literature suggests that when IrAEs are suspected, the best management approach is to consult a specialist to rule out infections and other more common causes of symptoms [7,9]. IrAE treatment relies on discontinuation of the offending agent and immunosuppressive therapy. The Common Terminology Criteria for Adverse Events (CTCAE) scale is used to grade this toxicity from 1-5 [10]. Grade I toxicity being the mildest does not necessitate discontinuation of immunotherapy [11]. Grade II toxicities, however, require delaying immunotherapy until symptoms and/or lab values improve to grade I, with the possibility of implementing steroid therapy if symptoms persist beyond one week [12]. Grades III and IV call for suspending immunotherapy and initiating high-dose intravenous steroid therapy. [11] Our case suggests that treatment with prednisone of 1 mg/kg until symptoms have been beneficial with discontinuation of the offending agents. Arterial embolization can also be considered if symptoms like hematuria do not improve with conventional management. However, larger studies are required to establish treatment guidelines regarding immunotherapy discontinuation, initiation of steroids, and interventional approach.

## Conclusions

Gastrointestinal and skin-related manifestations are considered the most reported IrAEs. However, the occurrence of non-bacterial cystitis in the urinary system, as reported in our case, is a relatively rare presentation. The occurrence of different IrAEs can vary between different ICI drugs and at different doses. The exact mechanism underlying these IrAEs remains unclear.
